# Increasing temperature and prey availability affect the growth and swimming kinematics of Atlantic herring (*Clupea harengus*) larvae

**DOI:** 10.1093/plankt/fbac014

**Published:** 2022-04-02

**Authors:** Bridie J M Allan, Howard I Browman, Steven Shema, Anne-Berit Skiftesvik, Arild Folkvord, Caroline M F Durif, Olav Sigurd Kjesbu

**Affiliations:** Institute for Marine Research (IMR), Pelagic Fish Research Group, PO Box 1870, Nordnes, Bergen 5817, Norway; Department of Marine Science, University of Otago, 310 Castle Street, Dunedin 9016, New Zealand; Institute for Marine Research (IMR), Ecosystem Acoustics Research Group, Austevoll Research Station, Sauganeset 16, Storebø 5392, Norway; Grótti ehf., Gundarstíg 4, Reykjavík 101, Iceland; Institute for Marine Research (IMR), Ecosystem Acoustics Research Group, Austevoll Research Station, Sauganeset 16, Storebø 5392, Norway; Institute for Marine Research (IMR), Pelagic Fish Research Group, PO Box 1870, Nordnes, Bergen 5817, Norway; University of Bergen (UiB), PO Box 7803, Bergen 5020, Norway; Institute for Marine Research (IMR), Ecosystem Acoustics Research Group, Austevoll Research Station, Sauganeset 16, Storebø 5392, Norway; Institute of Marine Research, Department of Marine Ecosystems and Resources, PO Box 1870, Nordnes, Bergen 5817, Norway

**Keywords:** climate change, fish larvae, zooplankton, food limitation, swimming behavior, recruitment

## Abstract

Climate change will increase the magnitude and duration of warming events and the variability in the phenology and abundance of available prey to the early life stages of fish. These factors influence physiological, behavioral and ecological processes, impacting growth, development and survival. Using a fully factorial design with two prey-availability treatments (1200 prey items L^−1^ (high prey abundance) or 40 prey items L^−1^ (low prey abundance)) under three temperature regimes (8, 10 and 12°C), the swimming kinematics of 6-week old spring-spawning Atlantic herring larvae were examined using silhouette video photography. Higher temperatures combined with food limitation significantly decreased the growth and swimming kinematics of larval herring, with the most negative effect observed in larvae reared at 12°C and exposed to low food abundances. Specifically, larvae displayed reduced locomotory behaviors and reduced vertical movements. By contrast, larvae reared at high prey abundance and at 12°C displayed more active swimming and exploratory behavior, as evidenced by an increase in both locomotory behavior and vertical and horizontal turn angles, suggesting increased motivation to search for food. This research highlights the importance of determining to what degree fish larvae are sensitive to changes in temperature and how these changes might be further influenced by food availability.

## INTRODUCTION

Sea surface temperatures (SST) are increasing (IPCC, 2019), impacting the physics and biology of marine environments in multiple ways. For example, increased thermal stratification and decreasing current strength are expected under warming scenarios (Sarmiento *et al*., 2004; Gattuso *et al*., 2015) with considerable impacts on the biodiversity and biogeography of marine taxa (Rosenzweig *et al.*, 2007; Doney *et al.*, 2012). Hence, these changes affect pelagic food webs, from phytoplankton to zooplankton to higher trophic levels, and may propagate along trophic interaction pathways resulting in cascading effects (Nagelkerken *et al.*, 2017). Ocean warming is predicted to be most pronounced in high-latitude, high-productivity seas such as the Barents and Norwegian Seas (IPCC, 2019). The Norwegian Sea and associated coastal areas support some of the world’s largest pelagic fish stocks such as blue whiting (*Micromesistius poutassou*), Atlantic mackerel (*Scomber scombrus*) and Atlantic herring (*Clupea harengus*) (FAO, 2020; Gullestad *et al.*, 2020).

Atlantic herring is, as the congener Pacific herring (*Clupea pallasii*), composed of numerous populations (dos Santos Schmidt *et al.*, 2021) that are principally defined by their spatio-temporal patterns of spawning (Geffen, 2009). The main commercial stocks of Atlantic herring are the Norwegian spring-spawning herring (NSSH), North Sea autumn-spawning herring and Icelandic summer-spawning herring (www.ices.dk). Herring are planktivorous and lay down eggs demersally (dos Santos Schmidt *et al.*, 2021). Their larvae are relatively robust to shortages of food (Folkvord *et al.*, 2015; Peck *et al*., 2020). In addition to the significant impact of predation (Bailey and Houde, 1989; Allan *et al.*, 2021), growth rates, development and survival are dependent on larvae finding sufficient quantities of food around the time that they deplete their yolk sac (Hufnagl *et al.*, 2015). The fact that some populations are spring spawners, and others summer or winter spawners, implies that the plankton community available to foraging herring larvae is highly variable and is dependent on season and locality. So, it is thus reasonable to assume that high winter temperatures (and, thereby, high metabolic costs, see below) resulting from global warming will be particularly detrimental for larvae of winter spawners, as indicated by marked increases in larval mortality observed at that time of the year (Hufnagl *et al.*, 2015).

Swimming ability changes rapidly during early ontogeny and is under strong selective pressure. For example, well-developed swimming ability can result in more effective dispersal and transport to nursery areas (Moyano *et al.*, 2016) as well as having other survival benefits such as efficient foraging (Munk and Kiørboe, 1985) and an increased ability to escape predators (Yin and Blaxter, 1987). Generally, the early life history stages (ELHS) have high metabolic demands and rapid growth rates (Brett, 1979; Oliver *et al*., 1979; Killen *et al.*, 2007). However, environmental changes that influence either the supply of energy, or the costs of cellular processes, will alter energy budgets, and consequently, individual performance (for reviews see Pankhurst and Munday, 2011; Illing *et al.*, 2020; Koch *et al*., 2021). For example, thermal variation not only affects an organism’s metabolic rates directly but also influences the interaction with other equally important variables such as prey availability. In this context, there is a need to characterize what the combined effect of reduced prey availability and elevated temperature will be on the growth and swimming kinematics of the larvae of marine fishes.

There is a negative interacting effect of higher temperature and lower food availability on growth in herring larvae (Folkvord *et al.*, 2009a, 2009b), but it remains to be documented if this is due to a higher energy expenditure caused by an increase in food search and swimming. The aim of this study was to determine whether elevated temperature and reduced prey availability would manifest as changes in overall growth, swimming performance and activity, important predictors of survival (McCormick *et al.*, 2018). We characterized this interaction in 6-week old Atlantic spring-spawning herring larvae using a fully factorial experimental design with two prey availability treatments (low and high abundance) at three temperatures (8, 10 and 12°C). Our design mirrored the reality that larvae experiencing different temperature regimes under climate fluctuations and change will be at different developmental stages at a given age (Trudgill *et al.*, 2005).

## MATERIALS AND METHODS

### Ethics statement

The Norwegian Institute of Marine Research’s Austevoll Research Station (IMR Austevoll) has a permit to operate as a Research Animal Facility for fish (all developmental stages), under Code 73 of the Norwegian Animal Welfare Act, Institutional Animal Care and Use Committee (IACUC). All animal handling and experimentation were approved by the Norwegian Animal Research Authority (NARA) (“Forsøksdyrutlaget”) (FOTS, ref. 15363) (https://www.mattilsynet.no/language/english/). After the trials, larvae were sacrificed using a humane endpoint sanctioned for fish.

### Larval rearing and collection

Local, spring-spawning herring were collected using a gill net at Askøy, Bergen, Norway (60.5°N, 5°E) on 8 March 2018 and used as the brood stock for production of the larvae used in this study. Naturally-hatched larvae—staged to be about 2 days old—from the same population were sampled on 23 March 2018, for measurement of larval standard length (*N* = 39) as detailed in Folkvord *et al.* (2020). This initial larval size and corresponding dry weight (Folkvord *et al.*, 2020) played the role as a reference point in the further evaluation of experimental larval growth.

These adults in question were transported to the Bergen High Technology Centre, UiB, where ripe males and females were strip spawned. Eggs from four females were stripped onto 12 glass plates in each of four separate seawater-filled trays with a water depth of 3 cm.

The gonads from five ripe males were removed and the sperm extracted. Sperm was activated and diluted with seawater before being added to the respective trays which created a total of 12 parental crosses. The use of multiple parental crosses was to ensure that there was enough genetic diversity present to accurately represent a population. As the mortality was generally low during the rearing, any phenotypic plasticity in terms of growth and size would be approximately equally represented in the different experimental groups (see below) and, thus, contribute to a higher confidence in the generality of the results. Egg plates were incubated at 10°C and 19 psu seawater for 30 min after which the plates were placed in 8°C and 35 psu seawater. After several minutes, each glass plate was transferred into a temperature-controlled room for incubation. This process yielded high-fertilization success (71–97%). Incubation tanks (2.2 m (length) × 0.4 m (width)) were supplied with filtered seawater at 8°C and at a rate of 5–10 L min^−1^.

Eggs were transferred to the nearby IMR Austevoll on 10 March 2018 in 5 L buckets filled with filtered and aerated seawater. There was no recorded mortality during the transfer period. Eggs plates were examined daily for mortality and dead eggs were noted and removed with forceps. Incubation tanks (50 L) were supplied with filtered seawater at a rate of 1.6 L min^−1^. Water was pumped from 160 m depth in the fjord outside IMR Austevoll and then sand-filtered and aerated. Temperature in the incubation tanks was kept at 8 ± 0.1°C. During the experiment, eggs were counted, and developmental stage was assessed on a degree day [(°*d* = temperature (°C) × time (*d*)] schedule (Trudgill *et al*., 2005). Using degree days, we estimated that the larvae were due to hatch on the 21st of March.

### Experimental treatments

Two days after hatch, larvae were transferred to 24 green fiberglass rearing tanks (50 L ~ 200 larvae in each) where they were split into six treatments, using four replicate tanks per treatment. Treatments consisted of ambient (8 ± 0.1°C—assumed to be a typical present-day coastal temperature (http://www.imr.no/forskning/forskningsdata/stasjoner/view/initdownload)–and elevated temperature groups (10 ± 0.1°C and 12 ± 0.1°C)). To avoid thermal-related shock, temperatures were raised by 1°C every 8 h until the final temperatures were reached (Allan *et al.*, 2017).

Larvae were fed a diet comprised initially of lipid enriched rotifers during the first 8 days post hatch (dph) which was followed by a diet of wild zooplankton collected using a wheel filter from a nearby saltwater pond (van der Meeren *et al.*, 2014). To accommodate for larval growth, the species and stage distribution of the copepods was changed during the experiment but were dominated by the calanoids *Eurytemora affinis* and *Centropages hamatus*. Food was provided twice daily at different, nominal prey abundances of either 1200 prey items L^−1^ (high prey abundance) or 40 prey items L^−1^ (low prey abundance). Prey values were based on previous research in which the 40 prey L^−1^ level has been shown to provide food-restricted growth, while 1200 prey L^−1^ represents *ad lib* food conditions for herring larvae at this stage (Folkvord *et al.*, 2000; Folkvord *et al.*, 2009a, 2009b). Each temperature treatment had a corresponding food treatment (high or low prey abundance). Algal paste (2 mL diluted in 100 mL seawater) was added to tank water to improve the contrast of the prey to aid in successful feeding (Illing *et al.*, 2015). Fluorescent light was delivered on a cycle of 14L:10D. Larvae were exposed to these treatments for six weeks. Hence, we focused on 6-week-old larvae, because mortality rates due to starvation (and predation) are exceedingly high in a field situation during the first 6 weeks of life but where the survivors show a fast increase in body mass (Folkvord *et al.*, 2020).

### Imaging system and experimental setup

Silhouette video photography (SVP) was used to observe the swimming behavior of the 6-week old herring larvae. The use of SVP to analyze the kinematics of behavior of fish larvae is preferable to conventional video imaging tools because it allows for high-quality observations of small, transparent organisms at high resolution and with a large depth-of-field, using low-intensity light sources to achieve the silhouette effect. The principles of the SVP system are described in detail elsewhere (Browman *et al.*, 2003). In brief, two video cameras are mounted orthogonally on optical rails with each camera illuminated by a 20-cm collimated beam generated by a small red (720 nm) LED light source. A glass aquarium holding the organisms is placed at the intersection of the two light paths. The two simultaneous orthogonal views allow particles in the field of view to be tracked in three dimensions. On the morning before each trial approximately 30 larvae were transferred into separate 20 × 20 × 20 cm observation tanks inside the SVP room. Larvae had not been fed that morning, with the last feed at ~6 p.m. the previous day. Therefore, we expect that the larvae had empty stomachs and were highly motivated to feed. No mortality was observed after transport.

The room temperature was adjusted using a heat pump system so that trials were conducted at the same water temperature from which the larvae originated. Larvae were acclimated for at least 2 h before each trial; one observation tank was used for each replicate rearing tank. Seawater from the rearing tanks was used to transport the larvae to the observation tank. The outer 2.5 cm of the observation tank was covered with black plastic contact paper, which restricted the field of view to the central 15 cm volume of water. This arrangement ensured that surface or edge effects did not affect the observations. A known amount of wild zooplankton (see above for zooplankton collection details) was added to the observation tank 10 min prior to recording to motivate foraging behavior. The prey density during the observations was about 3000 prey L^−1^ and was selected based on previous research investigating foraging behavior in cod (*Gadus morhua*) larvae using the same experimental system (Vollset *et al.*, 2011). All trials were conducted between the 26–29 April 2018 and the recording sequence of replicate tanks was randomized across the treatments. The total recording time for each replicate was 20 min with a temporal resolution of 60 frames per second (fps). After the trials, an additional 10 larvae per treatment group were added to the observational group of 30 larvae and sacrificed using an overdose of MS222 (ref. 15363, see above), digitally imaged, rinsed with milliQ water and dried in an oven at 40°C after which they were weighed.

### Video analysis

The software packages TrakFish, ManTrack and Anapaths (Racca Scientific Consulting and JASCO Research Ltd, Victoria, British Columbia, Canada) were used to analyze the video recordings (for full details, see Browman *et al.*, 2003). In brief, individual larval swim paths were reconstructed by combining the two orthogonally-recorded videos of each observation tank using the TrakFish software. The two videos were calibrated in TrakFish by creating a reference volume from known coordinates recorded in each view at the beginning of data collection. This reference volume establishes a scaled coordinate system from which the 3D spatial coordinates of larval location could be derived. Swim paths were then reconstructed in three-frame incremental steps (20 steps s^−1^) from the middle 5 min of the observation period to provide representative sampling.

Reconstructed paths were analyzed using Anapaths. Paths that were closely adjacent and resembled a single swim path were joined. Paths that remained too short (less than two body lengths) and/or had unrealistic swim trajectories were not included in the analysis. Individual swimming paths were then broken down into their component kinematic variables: move duration, distance and speed, stop duration, and horizontal and vertical turn angles (the change in direction after a stop in the horizontal and vertical planes). Stops were defined as movement of less than 1.5 mm s^−1^ (i.e. stationary/very slow coasting). The kinematic data points from each swim path were then pooled together within each replicate observation tank, meaning that individual larvae were not differentiated in the statistical analysis, since the independence of each path in the tank cannot be verified throughout the filming period.

### Statistical analysis

The effects of prior food availability (low and high) and elevated temperature (fixed factors) on growth was tested for the herring larvae using two-factor ANOVAs. Tukey’s HSD *post hoc* tests were then performed to determine the nature of any differences found by the ANOVA. Assumptions of normality and homogeneity of variance were examined using residual analysis and Levene’s test. The dry weight of the larvae did not meet the assumption of normality, so we performed a square-root transformation to normalize these records. Swimming kinematics were analyzed using the following variables extracted from the paths: move and stop durations, speed, path length, horizontal and vertical turn angles. The resulting data were visually inspected for outliers that corresponded to unrealistic move speeds (erratic behavior of larvae) and/or damaged larvae. Filtering out these outliers resulted in removing move speeds >40 mm s^−1^ and stop durations >25 s, representing a removal of 1% of the data. Distributions of the linear swimming kinematics variables were typically skewed towards lower values. Therefore, the data were log-transformed (*x* + 1). Swimming kinematics were analyzed using a linear model with combinations of food and temperature treatments as one variable. Pairwise comparisons were performed using the Tukey method. Models were fit in R and least-squares means were calculated using the R package “emmeans.” All residuals were normally distributed.

Circular statistics were computed for the turn angles. Since distributions of turn angles are typically skewed towards lower values (cf. above), we calculated the median instead of the mean, as well as skewness (to assess lack of symmetry) and kurtosis (which assesses the “tails” of the distributions or the range of the data). The common median direction between samples was tested using Fisher’s nonparametric test (Pewsey *et al*., 2013) and the circular homoscedasticity was tested using the nonparametric Wallraff test (Wallraff, 1979; Pewsey *et al.*, 2013).

## RESULTS

**Fig. 1:**
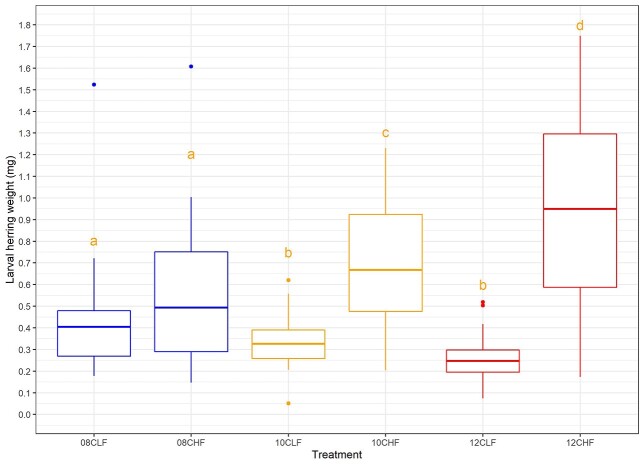
Dry weight (mg) (means ± SE) of herring (*C. harengus*) larvae reared under different temperature and food conditions (8, 10 and 12°C, low (L) and high (H) prey abundance). *N* = 40 in each group. Letters represent significant differences (different letters *P* < 0.05).

The dry weights of the 6-week-old larvae were significantly affected by the combined exposure of food availability and high temperature (*F*_(1, 236)_ = 42.91, *P* < 0.0001, Fig. 1) as well as temperature (*F*_(1, 236)_ = 7.56, *P* < 0.001, Fig. 1) and food (*F*_(1, 236)_ = 125.55, *P* < 0.001, Fig. 1) when considered separately. The heaviest larvae occurred in the high food and 12°C treatment, and the lightest in the low food and 12°C treatment. The noticed dynamic range (~0.2–1.5 mg) in final dry weights implied that those larvae with extreme low weights (~0.2–0.3 mg)—first of all found in experimental groups 10CLF and 12CLF (Fig. 1)—hardly had taken on any body mass, as the consulted reference material of 2-day old yolk-sac larvae showed a standard length of 9.16 mm (SD = ±0.52 mm) and thereby a typical dry weight of ~0.2 mg (Folkvord *et al.*, 2020).

**Fig. 2:**
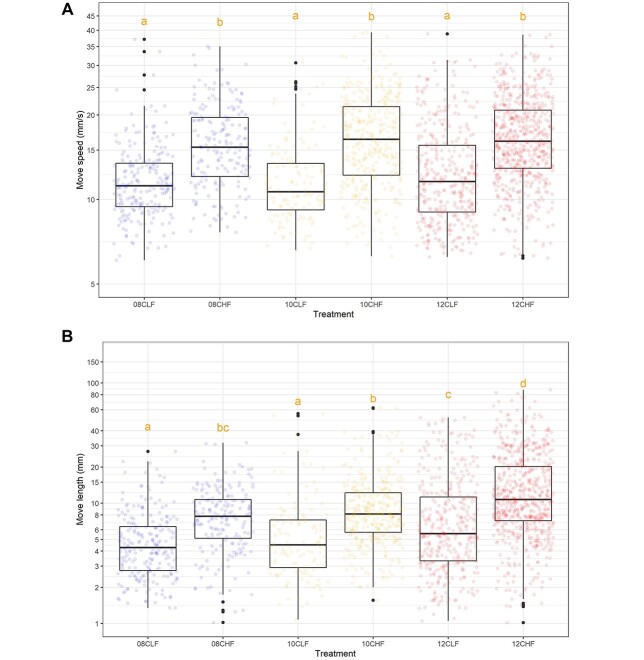

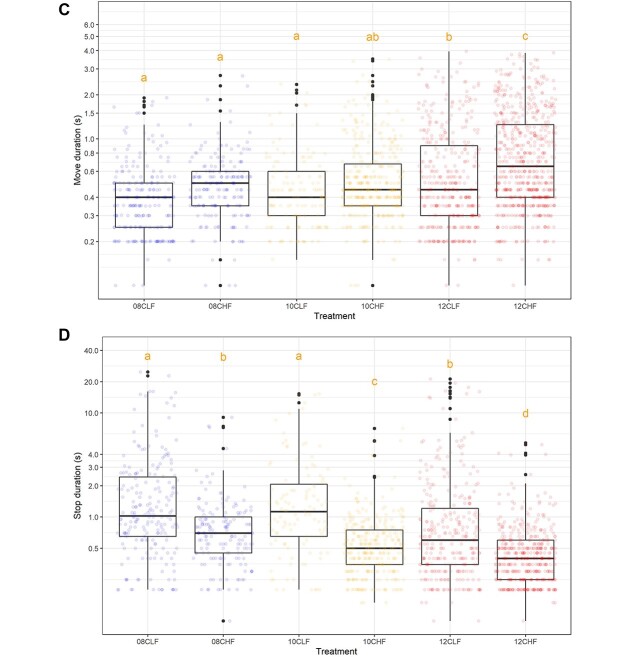
Comparison of the swimming kinematics of herring larvae (*C. harengus*) reared under different food and temperature conditions (8, 10 and 12°C, low (L) and high prey (H) abundance) and tested under high prey abundance. Values are summarized as boxplots and represented in the background using a jitter. Individual values are represented as dots. *Y*-axes are presented as log-scales. Letters represent significant differences (different letters *P* < 0.05).

**Fig. 3:**
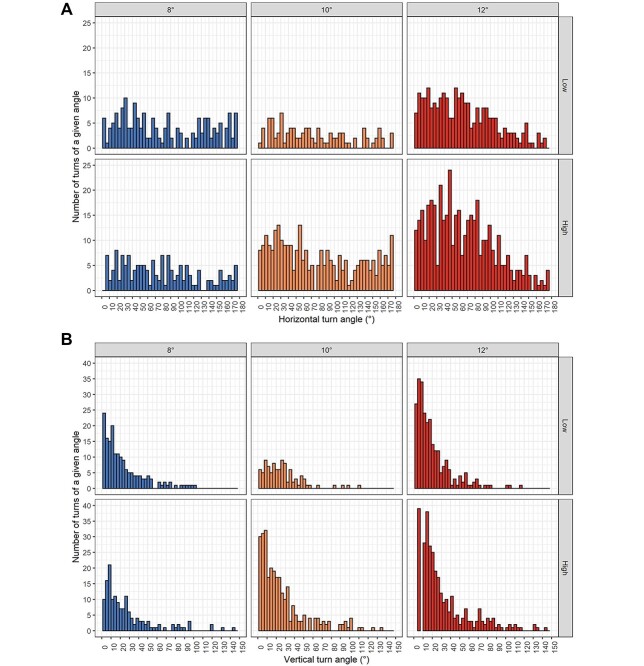
Swimming kinematics of herring larvae (*C. harengus*) reared under different food and temperature conditions (8, 10 and 12°C, low and high prey abundance) and tested under high prey abundance. The upper panel refers to number of turns in relation to the horizonal plane, whereas the lower panel in relation to the vertical plane.

**Fig. 4:**
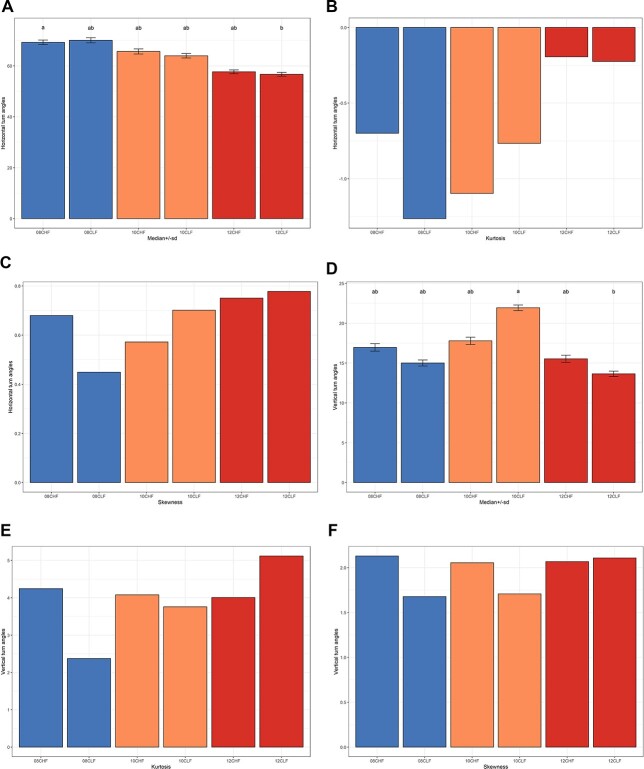
Circular statistics computed on swimming kinematics (turn angles in degrees) of herring larvae (*C. harengus*) reared under different food and temperature conditions (8, 10 and 12°C, low and high prey abundance) and tested under high prey abundance.

All swimming kinematics parameters consistently indicated increased activity of herring larvae from the high food treatment, and high temperature accelerated these differences (*P* < 0.05, Fig. 2). The increase in temperature from 8 to 10°C did not significantly affect behavior but so at 10 to 12°C. Move speed was the only parameter that was not significantly affected by temperature (*P* > 0.05) (Fig. 2). The larvae swam more actively (higher number of turns) at higher temperatures (Fig. 3). Circular variances of horizontal and vertical turn angles were significantly different according to treatment (*P* < 0.001), and median values of horizontal turn angles decreased with increasing temperature (Fig. 4). The latter outline was, however, only statistically significant between 8 and 12°C for larvae that had been reared in low prey abundance (*P* = 0.01, *P*_g_ 5.95) (Fig. 4). Diverging patterns were also apparent when examining skewness and kurtosis, which were higher at higher temperatures indicating fewer outliers and a more skewed distribution towards lower values. Observations were similar for vertical turn angles although not as clear (Fig. 4). Median values were only significantly different between 10 and 12°C at low prey abundance (*P* = 0.01, *P*_g_ 6.09). Skewness and kurtosis were higher at higher temperatures but only in the low food treatment (Fig. 4).

## DISCUSSION

Elevated temperature, coupled with food abundance, significantly affected the growth and swimming kinematics of spring spawning herring larvae. As expected, larvae reared in the low food abundance treatment exhibited lower growth relative to larvae reared in the high food abundance treatment. This effect was further magnified when decreased food abundance was coupled with increased temperature. Hence, larvae reared at 12°C and low food abundance had the lowest overall growth. Increasing temperature reduces the conversion of energy to somatic growth by increasing metabolic demand (Freitas *et al.*, 2010; Fouzai *et al*., 2015), particularly when prey availability is limited. Further, swimming behavior is affected by temperature owing to changes in growth rates (larger larvae swim faster), aerobic activity, endurance, cardiac output, as well as power output for anaerobic swimming (burst) through changes in the contractile properties of the swimming muscles (Fuiman and Batty, 1997; for review see Domenici *et al.*, 2019). Finally, the smaller the larvae, the lower the Reynolds number environment that they operate in, which affects the amplitude of forward propulsion during swimming and its energetic cost (Fuiman and Batty, 1997).

In the present study, the effect of temperature on swimming behavior only became significant from 10 to 12°C. Larvae reared under a high food and 12°C temperature regime displayed more active swimming and exploratory behavior, as evidenced by an increase in both locomotory behavior and vertical and horizontal turn angles. Although herring larvae are tolerant of periods of low food availability (Folkvord *et al.*, 2015), the increase in activity displayed by larvae reared in the high food and 12°C treatment group suggests an increased motivation to search for food. Yin and Blaxter (1987) suggest that swimming performance is only compromised after larvae reach the point of irreversible starvation (point of no return—PNR). We observed decreases in swimming behavior in fish reared under low food availability. Specifically, larvae displayed reduced locomotory behaviors and reduced vertical movements and this decrease in swimming behavior was more pronounced when combined with being reared at 12°C. It is also possible that the apparent decrease in swimming activity at the experimentally high prey densities may be the result of the larvae, reared under low food availability, attempting to stay in areas with high food abundance. Alternatively, it is possible that warming and decreased food availability led to changes in aerobic scope caused by an increase in resting metabolic rate due to exposure to elevated temperatures (Kiørboe *et al.*, 1987; Munday *et al.*, 2017). Fishes with reduced aerobic scope in under these conditions may therefore show reduced swimming behavior as an energy-saving strategy (Johansen and Jones, 2011). Analogous results have been observed for larval anchovy (*Engraulis mordax*) when encountering prey patches. For example, Hunter and Thomas (1974) demonstrated that larval anchovy reduced swimming speeds and increased changes in direction, both behaviors that increase time and search effort in a patch of food (see below).

Foraging success largely depends on the distribution of prey and the search strategy employed to find them (O'Brien *et al*., 1989). For example, when an animal encounters a patch of food, its foraging behavior is characterized by slower swimming speeds and larger turn angles, in a behavior referred to as Area Restricted Search (ARS) (Sommerfeld *et al*., 2013). Given the patchy nature of food resources in nature, this behavioral strategy is advantageous (Munk and Kiørboe, 1985). Animals also respond to decreasing prey abundance by increasing foraging time or decreasing activity as an energy saving strategy (for review see Sogard and Olla, 1996; Faria *et al*., 2011; Moran *et al*., 2021). In herring larvae, slow vertical sinking movement is associated with searching for prey while horizontal movement is associated with displacement into previously unsearched water (Browman and Skiftesvik, unpublished data). It is important to note that differences in growth, and thereby size-at-age, between larvae in the different groups may be partly responsible for the observed differences in swimming behavior at a given age and that the cumulative effect of reduced swimming behavior may be exacerbated in slower growing groups due to prolonged periods at smaller sizes and less developed stages (Stenevik *et al.*, 2021). The prolonged stage duration associated with slower growing individuals can result in elevated overall mortality in (Takasuka *et al.*, 2004). Further, herring larvae reared at warmer temperatures reach developmental milestones faster than those reared at lower temperatures and this will also have been partly responsible for the differences observed here (Moyano *et al.*, 2016; Stenevik *et al.*, 2021).

Many previous studies have focused on the effect of prey availability for the early life history stages (e.g. match/mismatch hypothesis (Cushing, 1990)) and how this can impact cohort strength (the so-called critical period (Hjort, 1914)). Such bottom-up processes dominate in shelf ecosystems such as along the Norwegian coast to the Barents Sea (Vikebø *et al*., 2012; Ferreira *et al.*, 2020) and in the Gulf of St. Lawrence (Brosset *et al*., 2020) and Gulf of Alaska (Laurel *et al.*, 2021) because of the large spatial heterogeneity and high complexity of such ecosystems (Fossum and Ellertsen, 1994). However, it is important to note that Atlantic herring are composed of numerous populations, each defined by their spatiotemporal patterns of spawning and exposure to different environmental regimes. Therefore, it is possible that each spawning population responds differently to warming and changing prey fields. For instance, Oskarsson and Taggart  (2010) observed a weakly positive link with Icelandic summer-spawning herring survival that was attributed to increasing temperatures associated with the North Atlantic Oscillation (NAO) winter anomaly. While the exact mechanisms of this could not be resolved, increased advection of larvae to favorable nursery grounds and enhanced vertical mixing increasing the availability of the naupliar stages of *Calanus finmarchicus* was suggested (Oskarsson and Taggart, 2010). However, most studies report decreased survival under conditions of warming and decreased prey availability, regardless of spawning pattern.

For example, Garcia *et al*. (2021) found in their review a decrease in recruitment with increasing temperature for NSSH larvae, and Payne *et al*. (2009, 2013) reported decreased survival in autumn spawning herring that was attributed to warming and decreased food quality/availability. In these respects, the long-term cyclicity (50–70 years) between cold and warm climate regimes—represented by the Atlantic Multidecadal Oscillation—is illustrative: in a cold regime there is a positive relationship between surface temperature and NSSH recruitment whereas this relationship turns to being negative in a warm regime (Tiedemann *et al.*, 2021). For fish larvae, a scenario of increased temperature and limited food availability is especially challenging given the high rates of growth and development that are characteristic at these early stages (for review see McMahon *et al*., 2019; Moran *et al.*, 2021). For example, from hatching to metamorphosis, Norwegian spring-spawning herring increase their body mass over 200-fold on their way to the Barents Sea (Folkvord *et al.*, 2020). Taken together, this complex interaction between temperature (warming), prey availability (phenology) and larval growth may dynamically shift—in either a positive or a negative direction—between energetic expenditures and energy gain, resulting in altered growth and performance which ultimately might affect recruitment dynamics. Such data would be useful to parameterize locomotory costs of herring larvae in individual based models that explore global change scenarios.

## CONCLUSION

Climate change is predicted to increase the magnitude and duration of warming events and potentially disrupt the phenology and abundance of available prey for larval fish. Using silhouette video photography, our findings demonstrate that higher temperatures combined with decreased prey availability significantly decreased the growth and swimming kinematics of larval herring, an important component of the North Atlantic ecosystem. These findings contribute to our understanding of how a warming environment and variability in the abundance of available prey may impact larval herring recruitment in future.

## DATA ACCESSIBILITY

Upon acceptance data and statistical code will be submitted to an online data repository. This information is available upon request during the review process.
